# Evaluation and implementation of multidisciplinary, standardized, guideline-based long-term follow-up care for adult survivors of childhood cancer in Germany: protocol of a prospective, multi-center, nationwide study (LE-Na)

**DOI:** 10.1186/s12885-025-14355-x

**Published:** 2025-05-22

**Authors:** C. Cytera, K. Baust, A. Borgmann-Staudt, G. Calaminus, K. Egger-Heidrich, J. Faber, D. Grabow, T. Halbsguth, A. Kock-Schopenhauer, I. R. König, S. Michaelis, A. Neumann, A. Puzik, S. Schuster, F. Wolters, C. Arendt, M. Sleimann, T. Langer, J. Gebauer

**Affiliations:** 1 Paediatric Haematology and Oncology, University Hospital of Schleswig-Holstein, Campus Luebeck, Luebeck, Germany; 2 Department for Paediatric Haematology/Oncology, University Hospital Bonn, Bonn, Germany; 3 Department of Paediatric Oncology and Haematology, Charité-Universitätsmedizin Berlin, Corporate Member of Freie Universität Berlin, Humboldt-Universität Zu Berlin, Berlin Institute of Health, Berlin, Germany; 4 Department of Internal Medicine I, University Hospital Carl Gustav Carus, Dresden, Germany; 5 Department of Pediatric Hematology, Oncology, and Hemostaseology, Center for Pediatric and Adolescent Medicine, University Medical Centerof the, Johannes Gutenberg-University Mainz , Mainz, Germany; 6 Division of Childhood Cancer Epidemiology / German Childhood Cancer Registry, Institute of Medical Biostatistics, Epidemiology and Informatics (IMBEI), University Medical Centreof the, Johannes Gutenberg University Mainz , Mainz, Germany; 7 Medical Clinic II, Haematology and Oncology, University Hospital Frankfurt Am Main, Frankfurt Am Main, Germany; 8 Institute of Medical Biometry and Statistics, Section for Clinical Research IT, University of Luebeck and University Hospital Schleswig-Holstein, Luebeck, Germany; 9 Institute of Medical Biometry and Statistics, University of Luebeck, University Hospital of Schleswig-Holstein, Campus Luebeck, Luebeck, Germany; 10 Department of Paediatric Oncology and Haematology, University Hospital Tübingen, , Tübingen, Germany; 11 Division of Paediatric Haematology and Oncology, Department of Pediatrics and Adolescent Medicine, Medical Center, Faculty of Medicine, University of Freiburg, Freiburg, Germany; 12 Department of Paediatric Haematology and Oncology, University Hospital Erlangen, Erlangen, Germany; 13 II.Department of Medicine, University Medical Center Hamburg-Eppendorf, Hamburg, Germany; 14 Department of Internal Medicine I, University Hospital of Schleswig-Holstein, Campus Luebeck, Luebeck, Germany

**Keywords:** Childhood cancer survivors, Long-term follow-up, Late effects

## Abstract

**Background:**

Late effects can occur years to decades after cancer therapy, resulting in morbidity and reduced health-related quality of life. Clinical long-term follow-up (LTFU) enables timely diagnosis and treatment of these sequelae. So far, only a minority of childhood cancer survivors (CCS) in Germany regularly visit LTFU care facilities.

The LE-Na study aims to: 1. implement and/or improve LTFU care structures for adult CCS in Germany, 2. inform former patients about late effects and LTFU care centers, 3. create a basis for future research by building up a central database, consent management and infrastructure, 4. establish a clinical LTFU cohort of adult CCS in Germany, 5. evaluate the implementation of the LFTU care, 6. enable the expansion of LTFU care structures nationwide, 7. integrate the developed LTFU care structures into the standard health care system.

**Methods:**

Within five years, approximately 5000 CCS will be invited to visit one of the 10 LTFU centers in Germany. Study participants are either contacted by the German Childhood Cancer Registry (GCCR), transitioned from the local pediatric oncology care unit, or recruited via media. They are assigned to one of three different risk groups based on an evidence-based risk stratification and receive standardized multidisciplinary follow-up care.

Primary outcomes are satisfaction with the LTFU care offer as well as degree of health-related self-efficacy expectation. They will be assessed at two time points. A scientific evaluation of the implemented LTFU care will be enabled by a waitlist control group. The harmonized outcome data are documented in a standardized database.

**Discussion:**

By addressing CCS in Germany who have not received standardized LTFU care yet, the LE-Na study expects to improve nationwide LTFU care and therewith patient’s satisfaction with the LTFU care offer as well as their health-related self-efficacy expectation.

## Introduction

During the last decades, cancer treatment improved continuously resulting in a growing community of childhood cancer survivors (CCS) worldwide [1]. As these survivors often face chronic health conditions, manifesting later in life as a consequence of the former cancer treatment, risk-adapted long-term follow-up (LTFU) is recommended to facilitate timely diagnosis and treatment of possible late effects [2]. However, only a few specialized survivorship care units have been established in Germany to date. Additionally, as most studies focusing on late effects of cancer treatment have been conducted during the last two decades, many CCS that were treated prior to 2000 have not been informed about their individual risk for late-effects nor have they been invited to regular LTFU [3, 4].

Consequently, the multi-centric, prospective LE-Na study has been developed in order to implement and evaluate multidisciplinary, standardized, guideline-based LTFU care for adult survivors of childhood cancer in Germany and thus improve survivorship care nationwide.

Following on from previous pilot studies, LE-Na pursues seven objectives [4, 5]. First, to expand multidisciplinary LTFU structures to improve detection and treatment of late effects as well as psychosocial challenges and mental health impairments in CCS. Second, to provide appropriate information about late effects and recommendations for LTFU care for CCS who were treated in the 1980s and 1990s as they may not have been informed about late effects yet. Third, to create a platform for future research by building up a central database, consent management and infrastructure. Fourth, to establish a clinical LTFU cohort of German CCS in order to prospectively gain knowledge on late effects. Fifth, to evaluate the implementation of the LTFU care. Sixth, to enable the expansion of LTFU structures nationwide by offering harmonized and evaluated procedures as well as documents to clinics that do not have a survivorship care unit yet. And seventh, allowing the integration of LTFU care structures as developed and evaluated in this project into the standard health care system.

## Methods

### Study design

**Fig. 1:**
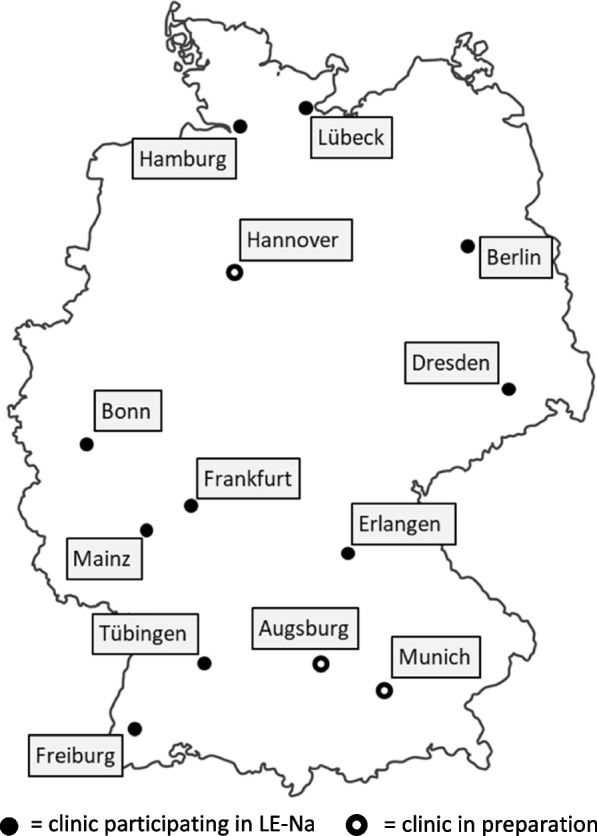
German LTFU clinics currently participating or in preparation to participate in the LE-Na trial. Lübeck – University Hospital of Schleswig–Holstein, Campus Lübeck. Hamburg—University Hospital Hamburg-Eppendorf. Berlin –University Hospital Charité Berlin. Hannover – University Hospital Hannover. Dresden—University Hospital Carl Gustav Carus, Dresden. Bonn—University Hospital Bonn. Frankfurt—University Hospital Frankfurt am Main. Mainz—University Hospital of the Johannes Gutenberg-University Mainz. Erlangen—University Hospital Erlangen. Tübingen—University Hospital Tübingen. Augsburg – University Hospital Augsburg. Munich—University Hospital Rechts der Isar, Munich. Freiburg –University Hospital Freiburg

LE-Na is a prospective, multi-center study which will be conducted in 10 LTFU clinics in Germany. Further clinics will be invited to participate during the study period as soon as they have set up a multidisciplinary LTFU team and follow the evidence-based risk stratification used in LE-Na [3, 6] (see Fig. 1).

The LE-Na trial was preceded by several feasibility studies and the components were constantly reevaluated [4, 5]. An external advisory board as well as a representative of German health insurance companies participate in study meetings and advise the study consortium. However, they do not have access to study data. Survivors representatives as well as health economic specialists have been included in study preparation but, due to organizational issues, do not participate in study meetings.

During the study period of five years (January 1st 2023 to December 31st 2027), primary outcomes will be assessed in a pre-post-comparison (outcomes and measurements see Table 1). Therefore, after giving consent, study participants will be asked to complete a set of questionnaires at the initial consultation at the LTFU clinic as well as after three years (phase 1). The first assessment always takes place at the clinic and is tablet-based; the second assessment after three years can either take place at the clinic during regular LTFU (participants from risk group 2 and 3, see also “standardized LTFU care and risk stratification”) or web-based/ on paper if the participant is not regularly attending the clinic in this year. The results of the survey, all examination results and medical history will be documented in a pseudonymous form in a clinical trial management system (CTMS) in order to enable a central and coherent recording of late effects [5].

**Table 1:** Primary and secondary study outcomes

Category	Instrument/measurement
**Primary outcomes**	
Satisfaction with the LTFU care offer	Patient Satisfaction Questionnaire-Short Form, PSQ-18^a^
Degree of health-related self-efficacy expectation	General Self-Efficacy-Scale (GSE)^a^
	Healthcare Self-Efficacy-Scale – (HCSE)
**Secondary outcomes**	
Implementation of guideline recommendations in specialized LTFU care centers	Operationalized as use of risk stratification and guideline recommendations by the study centers (annual survey)
Health related quality of life	EORTC QLQ C30
Incidence and prevalence of late effects	

^a^Instructions of the PSQ-18 and GSE have been slightly adapted to set the questionnaire into the context of LTFU after childhood cancer

Additionally, during study year 3 and 4 (phase 2), the implementation of LTFU care will be tested randomized using a waiting list control group, again, regarding the primary endpoints. This intervention was developed together with health economists in order to provide evidence for the benefit of standardized LTFU care that can be used to obtain full cost coverage by the health insurance companies. All potential study participants in this phase are contacted by the GCCR and are asked to participate in this randomized trial. Randomization is carried out by the GCCR that defines a group of potential participants that is stratified according to gender, cancer diagnosis, time since end of therapy and residency. Participants of the intervention group in phase 2 receive an invitation to visit the LTFU clinic within the following three months. They complete the questionnaires web-based before their first visit as well as after 12 months. Participants of the waiting control group receive an invitation to complete the questionnaires web-based initially and a second time after 12 months. After filling out the questionnaire at these two time points, the waiting control group visits the LTFU clinic (one year later than the intervention group) and receives the same standardized LTFU care.

### Study population

Currently, about 1000 patients visit one of the LTFU care centers in Germany per year, which corresponds to about 3% of adult CCS in Germany. The LE-Na study aims to bring additional 5000 survivors into regular LTFU care through three different recruitment strategies:

1. Regular transition from the pediatric care unit
2. Invitation to participate in the LE-Na study sent out to 5000 CCS by the German Childhood Cancer Registry (GCCR) [7], fulfilling the following criteria
  initially treated for childhood cancer between 1980 and 1999
  Living within a radius of 50–100 km from one of the study centers
3. media, e.g. flyer, survivor meetings, project homepage, social media

Inclusion criteria for the LE-Na study are:

- 18 years or older at the time of study inclusion
- cancer diagnosis and therapy in childhood or adolescence (before 18.^th^ birthday)
- completed regular oncological follow-up care (at least 5 years after end of treatment of the primary oncological disease)
- in case of relapses or secondary neoplasia: at least 6 months after completion of radiotherapy/chemotherapy, at least 12 months after completion of stem cell transplantation
- good understanding of the German language

Exclusion criteria of the LE-Na study are:

- people not capable of giving consent
- already received interdisciplinary LTFU care
- current oncological therapy

### Ethics approval and consent to participate

This study was approved by the ethics committee of the University of Lübeck (registration number 2022–647). Given informed consent is required to participate in the study.

Potential participants in phase 1 will be informed about the study prior to the initial consultation and, if interested, asked to give their written informed consent by the physician in charge as soon as they visit the LTFU clinic. Participants document on the informed consent form whether they want to be contacted via e-mail or mail to fill out the second questionnaire after three years if they do not attend the clinic at this time during their regular LTFU.

Potential participants of the waiting list control design (phase 2), will be invited to take part in the study via mail by the GCCR. The invitation contains information about the random allocation to the intervention or the waiting control group and explains that the randomisation result will be communicated at a later date. If CCS are interested to participate, they contact a LTFU clinic of their choice by telephone. Each LTFU center has an ID list of all CCS contacted by the GCCR along with the randomisation results. The respective LTFU center coordinator asks the CCS if they confirm study participation in the randomization trial. If the CCS agrees, the LTFU center coordinator records the CCS's contact data and informs the CCS afterwards by email about the randomisation result and a proposed date for the first LTFU appointment. The participant also receives a link to a portal (e.g. SoSci Survey) to fill in the questionnaires web-based before the first consultation in the clinic. Prior to answering study items, participants have to read the study information and give their consent by ticking the regarding consent-statement. They also have to fill in their study ID, provided in the invitation letter sent by the GCCR, to identify themselfes. The follow-up survey after one year will also be sent to the participants via the respective centres (as in phase 1). CCS who are interested in LTFU care but refuse to participate in the randomisation trial before they were informed about the randomization result are asked to participate in the pre-post-comparison (phase 1).

### Data collection and measurements

CCS who transition regularly from pediatric care, will be informed about interdisciplinary LTFU care by their treating pediatric oncologist. This information is communicated up to one year before the transition to allow the CCS to adapt to the upcoming change. A few weeks before the first LTFU consultation, the CCS will be informed about the LE-Na study according to local standards, e.g. via telephone plus additional information via e-mail.

CCS registered in the GCCR receive a letter with study information as well as a list of all LTFU clinics which are part of the LE-Na project. When interested, they contact a clinic nearby, may ask questions about LTFU and arrange a date for the consultation.

**Fig. 2:**
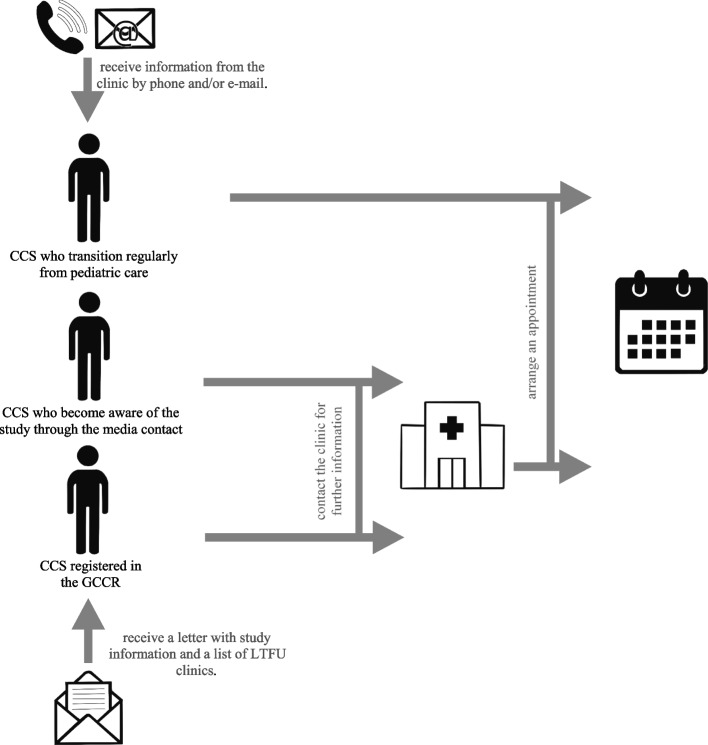
Recruitment strategies for the LE-Na study

CCS who become aware of the study through the media contact the center of their choice directly, have the opportunity to receive further information and arrange an appointment (see Fig. 2).

On the day of the consultation, CCS will first be welcomed by the LTFU team and receive information about the study from a designated team member, e.g. the study coordinator or psychologist. If participation in the study is desired, patients sign the consent form and receive a tablet on which they fill out the study questionnaires and those of the psychosocial screening. The completion duration is around 20–30 min. This is followed by a consultation with the LTFU care doctor and all the necessary examinations (e.g., ECG, MRI), which were planned beforehand based on the previous oncological disease and treatment exposure (risk stratification [6], see “Standardized LTFU care and risk stratification” below). If examinations are not available at some centers, patients are advised how to coordinate a separate appointment. In these cases, the corresponding LTFU care center collects these additional data and documents them in the database afterwards. Thereafter, the patient is introduced to the psychosocial expert, who explores the psychosocial history based on the psychosocial screening and identifies any psychosocial difficulties (see “ Psychosocial screening” below). After case discussion in the interdisciplinary team, the results of the examinations and expected long-term consequences are communicated with the patient, who thereby receives an individualized LTFU plan. After the consultation, all examination results and the patient's medical history are documented in a CTMS; the completed questionnaires (both for evaluation and for psychosocial screening) are transferred automatically to the CTMS (see “ Data processing and data quality” below).

The waiting control group (study phase 2) receives the standard care provided by their general practitioner during a one-year waiting period. The extent of this individual use of preventive examinations before participation in the study is assessed in the studies questionnaire.

The instruments to assess the study outcomes are summarized in Table 1.

In addition to measuring the displayed outcomes, several secondary analyses will be carried out:

- Gender differences in study recruitment strategies – It will be investigated whether different recruitment strategies result in differences in the gender distribution among study participants. Therefore, the study centers document in the CTMS how each participant became aware of the study: Either through transition, the GCCR or media.
- Sensitivity of the questionnaire-based psychosocial screening compared to the interview-based psychosocial anamnesis – This will be operationalized as the number of cases that receive a psychosocial follow-up appointment due to the interview-based psychosocial history, although the cut-off values of the questionnaire-based psychosocial screening were not exceeded.
- An interim analysis after one study year is going to be performed to identify problems in study recruitment (with regard to the three different recruitment strategies) as well as possible underrepresentation of certain survivor groups (for example survivors with migration background). For this analysis, all survivors that do not meet the inclusion criteria or meet one exclusion criterion are documented in each center anonymously (age, gender, primary cancer, exclusion / inclusion criterion (not) met). Also, a non-responder analysis of those survivors that are contacted via the German childhood cancer registry is going to be conducted. Adaptations in recruitment strategies in order to engage study participation are going to be discussed based on this interim analysis.

### Standardized LTFU care and risk stratification

Standardized LTFU care is performed by an interdisciplinary team consisting of a core team (specialists for pediatric oncology and internal medicine, psychosocial expert and a case manager) and further organ specialists (e.g. neurologists, dermatologists). It is based on a compilation of international evidence-based guidelines from the International Guideline Harmonisation Group (IGHG) and PanCare that have been summarized in 2020 and updated in 2023 [3, 6]. This compilation includes examinations for early detection of possible late effects primarily aiming at asymptomatic survivors. All participating clinics have experience with LTFU for CCS and have all necessary resources to perform standardized LTFU care which was independently reviewed prior to the study initiation by an external advisory board consisting of 4 survivorship specialists from Germany, Austria and Switzerland.

During LTFU, a previously published definition of three different risk groups based on cancer treatment exposure is used to stratify CCS according to their future risk to develop late effects [6, 8]. In this risk stratification, risk group 1 (RG1) includes CCS with a low risk for late effects. This group comprises CCS who were treated with surgical therapy (except CCS with a central nervous system (CNS) tumor), survivors of acute lymphoblastic leukemia and patients with non-hereditary retinoblastoma who received chemotherapy only. Risk group 2 (RG 2) includes CCS with an intermediate risk for late effects, such as survivors following chemotherapy only (excluding CCS assigned to RG1) or survivors of CNS tumor who had undergone surgical therapy. CCS with the highest risk for late effects are summarized in risk group 3 (RG3) such as survivors after hematopoietic stem cell transplantation and/or radiotherapy [5]. CCS return periodically to the clinic depending on their RG (RG 1: every 5 years, RG 2: every 2 to 3 years, RG 3: yearly) and continuously receive LTFU care.

### Psychosocial screening

Psychosocial screening questionnaires are presented via tablet following those questionnaires to assess primary and secondary outcomes. They focus on (mental) health impairments which have been identified as relevant for survivors after childhood cancer [9, 10] and includes widely established questionnaires which have been implemented in former prevention studies recruiting childhood cancer survivors in Germany ( [11] see Table 2). In order to adapt the screening procedure for survivors with cognitive impairments, a short version is offered including the Distress Thermometer without additional questions on daily life issues, the PHQ-4 and a single question on posttraumatic symptoms. First, all CCS start with the same questionnaires and are asked to complete the long form, if possible. However, if they feel tired or exhausted, they can choose to switch to the short form after filling out the FA 12 (see Table 2). This approach is going to be evaluated in an interim analysis after one year. Psychosocial experts are able to retrieve an individual summary of results with guidance on interpretation and cut-off via CTMS. They are encouraged to include screening results into the psychosocial anamnesis, focusing on those symptom areas patients indicated score above the cut-off. This is especially relevant in those patients who completed the short form of the screening or who indicated suicidal ideation in the PHQ-9 in the long form. For the same reason LTFU teams are advised not to offer the psychosocial screening if psychosocial experts or physicians experienced with mental health impairments are not available during the consultation day and cannot follow- up the screening results face-to-face. In order to provide a harmonized approach and improve quality assurance regarding the psychosocial screening, psychosocial experts are invited to participate in regular intervision meetings.

**Table 2:** Questionnaires used in the psychosocial screening in LE-Na

Long Form Psychosocial Screening	Short Form Psychosocial Screening
EORTC QLQ C30 [12]	EORTC QLQ C30 [12]
EORTC QLQ FA12 [13]	EORTC QLQ FA12 [13]
Distress Thermometer with problem areas [14]	Distress Thermometer without problem areas [14]
PHQ-4 [15]	PHQ-4 [15]
PHQ-9 (last seven questions) [16]	
IES-R [17]	Screening question PTSD [9]
Questions on life situation and educational & occupational outcomes	Questions on life situation and educational & occupational outcomes

### Data processing and data quality

All examination results and the patient's medical history are documented in a CTMS after the patient's consent has been obtained [5]. The documentation takes place every time the patient attends the clinic during the study period (e.g. yearly for CCS assigned to RG 3, see also “standardized LTFU care and risk stratification”) although information on primary cancer diagnosis and treatment are only filled out at first data entry. This enables a central and uniform recording of chronic health conditions in CCS and facilitates the subsequent evaluation of the study. Each center has its own area to document the subjects pseudonymously. In total, the study consists of 15 case report forms (CRFs). 10 CRFs are filled out by the subjects themselves using a tablet (for further information about the database, see [5]). Depending on the risk group and the respective study phase, the corresponding CRFs are displayed on the tablets and completed by the study participant. The completed CRFs are then transferred to the CTMS. The study coordinator ensures that the assignment was carried out correctly. Data management includes more than 50 validation rules and further plausibility checks. Intermediate evaluations are planned to monitor data quality.

### Statistical analysis

With regard to the pre-post-comparison after three years in phase 1, small to medium effect sizes can be assumed based on the literature. However, it must be considered that the largest effects are expected in the stratum (i.e. in the group) of patients with high and medium risk, and smaller effects in the stratum of patients with low risk. Therefore, the two strata with high and medium risk are first tested equally, and the stratum with low risk is tested separately in a hierarchical manner. In order to comply with the global significance level of 5% when testing initially two strata and two primary endpoints, a local significance level of 1.25% is used in each case. With a power of 90%, a sample of *n* = 370 per stratum is sufficient to detect an effect of delta = 0.2. This number is achieved in the strata with medium and high risk if 1000 patients are included per year, of whom 400 patients fall into the group with high and medium risk respectively and of whom about 95% participate in the study. Patients enrolled in the first year will be surveyed in the fourth year with a 3-year follow-up, at which time the primary outcome criteria will be measured.

Regarding the parallel group comparison in phase 2, the sample size is estimated so that an effect of delta = 0.25 can be detected with a power of 80% for both primary endpoints. 310 patients per risk group (each in the intervention and control group), i.e. 620 patients in one stratum, must therefore be included in the study. If the participation rates in the first two years of the study (captured in an interim evaluation after two years) already indicate that the number of probands for the summative evaluation will be too low despite the recruitment measures described, only one single endpoint can be analyzed in the final evaluation. In this case, 255 patients per group in one stratum, i.e. 510 patients in one stratum in total, would be included in the study.

In both phases, the primary analyses will be performed by estimating generalized linear models including primary oncological disease, sex, and time since treatment as covariables. Details will be fixed in the statistical analysis plan.

## Results

The LE-Na study started in January 2023 and inclusion of eligible patients transitioning from pediatric to adult care has begun in October 2023 after all requirements where met (e.g. signed contracts, respective team members participated in CTMS- and psychosocial training). Recruitment via GCCR started in February 2024. Inclusion of participants and data collection will continue until the end of 2027.

## Discussion

As many CCS are affected by chronic health conditions later in life that may occur as a consequence of former cancer treatment exposure, life-long LTFU care is essential to ensure timely diagnosis and treatment of possible late effects [2, 18]. Several guidelines and expert groups propose a center-based care model with multidisciplinary LTFU teams as central coordinator of LTFU care [19–21]. These centers offer experience and knowledge about late effects and provide risk-adapted surveillance examinations. Additionally, information about late effects of cancer treatment as well as advice on a healthy lifestyle as a preventive measure can be integrated in the consultations [6]. However, only a few centers in Europe offer LTFU care for adult CCS so that continuation of care after transition from pediatric to adult care unit is often interrupted [19]. Consequently, the majority of adult CCS in Germany are currently not in specialized LTFU care as they have either never been informed about their potential risk to develop late effects (being treated in the 1980s, 1990s or even earlier) or finished LTFU care after end of surveillance in the pediatric oncology department as no corresponding specialized adult centered care unit was available.

In order to improve LTFU care for adult survivors of childhood cancer in Germany, the LE-Na study was developed aiming to include 5000 CCS that have not received LTFU care yet. Therefore, national LTFU care structures have been established prior to study initiation at ten university hospitals providing standardized and risk-stratified LTFU care for this patient cohort. It is intended to expand this network of clinics. Standardized and risk-stratified LTFU care, as described in this manuscript, is currently already being set up in three additional clinics. Evaluation of this care offer, looking at different endpoints such as satisfaction with the LTFU care offer as well as degree of health-related self-efficacy expectation, will be used to facilitate adaptions of care structures to patients’ needs.

One of the largest German health insurance companies is involved in an observational capacity in the study to advise on continuation of care in the German health care system. Although individual examinations (e.g. echocardiogram) are already fully covered by the German health care system, financing of comprehensive LTFU care programs including psychosocial care in accordance with current guidelines, as performed in this trial, is still largely lacking. The results of the LE-Na study as well as documentation of the prevalence of clinically validated chronic health conditions in the largest cohort of long-term German CCS studied so far will help to develop cost coverage models to ensure continuation of care after the end of the study period.

A common platform for future research along with the establishment of a defined long-term cohort of German CCS gives the possibility to follow-up these optimizations and, ultimately, may allow the integration of the developed and evaluated LTFU care structures into the standard health care system.

## Data Availability

The data sets used in the current study are available upon reasonable request from the corresponding author.

## Abbreviations

CCS: Childhood cancer survivors
CNS: Central nervous system
CRF: Case report form
CTMS: Clinical trial management system
GCCR: German Childhood Cancer Registry
GSE: General Self-Efficacy-Scale
HCSE: Healthcare Self-Efficacy-Scale
LE-Na: Evaluation und Implementierung einer multidisziplinären, standardisierten, leitliniengerechten Langzeitnachsorge für heute Erwachsene, ehemals krebskranke Kinder und Jugendliche, in Deutschland – Eine prospektive, multizentrische, bundesweite Versorgungsstudie im Nachsorgenetzwerk (German study title)
LTFU: Long-term follow-up
RG: Risk group
